# Sortilin‐1, Targeted by miR‐146a, Regulates the Behavior of Non‐Small Cell Lung Cancer

**DOI:** 10.1111/1759-7714.70129

**Published:** 2025-08-09

**Authors:** Xi Lin, Zhi Yan, Ling Hai, Yan Niu, Jian‐Xun Wen, Hong‐Zhe Zhu, Cheng Yan, Su‐Na Cha, Li Yan, Wen‐Qi Zheng, Man Zhang, Zhi‐De Hu

**Affiliations:** ^1^ Department of Thoracic Surgery the Affiliated Hospital of Inner Mongolia Medical University Hohhot China; ^2^ Department of Laboratory Medicine the Affiliated Hospital of Inner Mongolia Medical University Hohhot China; ^3^ Key Laboratory for Biomarkers Inner Mongolia Medical University Hohhot China; ^4^ School of Medical Laboratory Tianjin Medical University Tianjin China; ^5^ Department of Pathology Basic Medical College, Inner Mongolia Medical University Hohhot China; ^6^ Department of Pathology the Affiliated Hospital of Inner Mongolia Medical University Hohhot China; ^7^ Public Service Center for Medical Research Inner Mongolia Medical University Hohhot China; ^8^ Department of Respiratory and Critical Care Medicine the Affiliated Hospital of Inner Mongolia Medical University Hohhot China; ^9^ Department of Thoracic Surgery and Oncology the Key Laboratory of Advanced Interdisciplinary Studies, National Center for Respiratory Medicine, National Clinical Research Center for Respiratory Disease, State Key Laboratory of Respiratory Disease, Guangzhou Institute of Respiratory Health, the First Affiliated Hospital, Guangzhou Medical University Guangzhou China; ^10^ Center for Clinical Epidemiology Research the Affiliated Hospital of Inner Mongolia Medical University Hohhot China

**Keywords:** miR‐146a, NSCLC, sortilin‐1

## Abstract

**Background:**

Sortilin‐1 (SORT1) has been implicated in the pathogenesis of various malignancies, but its role in non‐small cell lung cancer (NSCLC) remains to be elucidated.

**Methods:**

Immunohistochemistry was employed to assess the expression of SORT1 in cancerous tissues compared to adjacent non‐cancerous tissues. NSCLC cell lines, including A549, H1299, and PC‐9, underwent treatment with miR‐146a mimics or SORT1 small interfering RNA (siRNA), followed by evaluations of cell viability, migration, invasion, and apoptosis using cell counting kit‐8, transwell assays, and scratch wound assays. Additionally, bioinformatic methods were employed to predict miR‐146a target genes, which were subsequently validated through dual‐luciferase reporter assays.

**Results:**

SORT1 was significantly elevated in NSCLC tissues compared to adjacent non‐cancerous counterparts. Downregulation of SORT1 inhibited proliferation, invasion, migration of tumor cell lines and promoted apoptosis. Moreover, SORT1 was a direct target of miR‐146a. MiR‐146a modulated tumor cell proliferation, migration, invasion, and apoptosis by suppressing SORT1 expression.

**Conclusion:**

These results suggest that miR‐146a plays a critical role in the pathogenesis of NSCLC by targeting SORT1, highlighting its potential as a therapeutic target for NSCLC.

## Graphical abstract

Immunohistochemistry revealed elevated sortilin‐1 expression in NSCLC patient tissues compared to adjacent normal tissue. In vitro, miR‐146a directly targeted and downregulated sortilin‐1 in NSCLC cell lines, as confirmed by dual‐luciferase reporter assay, qRT‐PCR, and Western blot. Silencing sortilin (via siRNA) or overexpressing miR‐146a (via mimic) significantly inhibited NSCLC cell proliferation (CCK‐8), migration (scratch/transwell), and invasion (transwell), while promot ed apoptosis (flow cytometry).

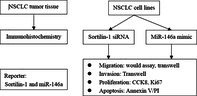

## 1 Introduction

Lung cancer is among the most prevalent malignancies globally and constitutes a principal cause of cancer‐related mortality [1, 2]. Approximately 85% of lung cancer cases are classified as non‐small cell lung cancer (NSCLC), which is associated with an inferior prognosis, evidenced by a five‐year survival rate of merely 15% [3]. Despite the emergence of novel treatment modalities for NSCLC, including immunotherapy and targeted therapy, which can moderately enhance patient outcomes, the overall prognosis remains disconcertingly poor. Notably, patients diagnosed with advanced‐stage disease exhibit an exceedingly low 5‐year survival rate of only 5% [4]. Consequently, there is an urgent imperative to investigate the underlying mechanisms governing lung cancer pathogenesis, thereby facilitating the identification of novel therapeutic targets.

Sortilin‐1 (SORT1) is a highly conserved protein initially identified in brain tissue that can bind to receptor‐associated protein (RAP) residues located on the endoplasmic reticulum and Golgi complex [5]. While SORT1 is predominantly expressed within the central nervous system, it is also markedly present in various peripheral organs and tissues. Its involvement in the pathogenesis and progression of a range of chronic diseases has been well documented, encompassing hyperlipidemia, diabetes mellitus, Alzheimer's disease, and malignancies [6, 7]. Notably, the overexpression of SORT1 has been observed in various tumors, including breast [8], ovarian [9], and thyroid [10] cancers. However, there remains a relative paucity of research investigating the specific relationship between SORT1 and lung cancer [11]. Current studies primarily examine the cellular aspects of SORT1 functionality, lacking comprehensive exploration into the underlying molecular mechanisms that mediate SORT1's specific biological roles and the regulatory frameworks governing its expression.

MicroRNAs (miRNAs) are a class of short, non‐coding RNAs that play a crucial role in regulating gene expression at the post‐transcriptional level. This regulation occurs through the binding of miRNAs to the 3′ untranslated region (3′UTR) of target mRNAs, resulting in their degradation or the inhibition of translation [12]. MiRNAs are widely implicated in various physiological and pathological processes, including embryonic development, wound healing, inflammatory responses, and tumorigenesis [13]. Recent studies indicate that miRNAs can significantly influence the initiation and progression of lung cancer, affecting both the tumor cells and the surrounding tumor microenvironment [14]. MiR‐146a has emerged as one of the earliest recognized miRNAs involved in the regulation of inflammatory responses [15]. In addition to its role in modulating immune responses, miR‐146a is also implicated in the development and progression of tumors [16, 17]. Evidence suggests that the expression of miR‐146a is downregulated in the tumor tissue of lung cancer patients [18, 19]. Moreover, miR‐146a may influence the development and metastasis of lung cancer by targeting critical signaling molecules, including Akt, p53, and RANTES [17].

The present investigation elucidated that SORT1 is significantly overexpressed in NSCLC tumor tissue. Downregulation of SORT1 substantially impairs the migratory and invasive capacities of NSCLC cell lines. Further mechanistic analyses demonstrated that SORT1 serves as a direct target of miR‐146a. Additionally, the data suggest that both SORT1 and miR‐146a play pivotal roles in modulating the migratory, proliferation, and invasive behaviors of NSCLC cells. Consequently, we conclude that miR‐146a attenuates the migration and invasion of NSCLC by directly targeting SORT1. To the best of our knowledge, this study represents the first comprehensive analysis of the functional and mechanistic interplay between miR‐146a and SORT1 and their influence on the tumor properties of NSCLC cells.

## 2 Materials and Methods

### 2.1 Immunohistochemistry

We collected tumor tissue specimens from 52 patients with NSCLC. Patients with other types of tumors were excluded. We used immunohistochemistry to detect the SORT1 expression in tumors and adjacent tissues. The mean age of the patients was 59 years, encompassing 28 males and 24 females. The ethics committee of the Affiliated Hospital of Inner Mongolia Medical University approved the study. The primary and secondary antibodies for SORT1 immunohistochemistry were from PTG (China, 12 369‐1‐AP) and Maixin (China, ABD‐0030). In addition, we also searched the Human Protein Atlas database to confirm our immunohistochemical findings (https://www.proteinatlas.org/, June 23, 2025).

### 2.2 Cell Culture

NSCLC cell lines (H‐1299, A549, and PC‐9) were purchased from BNCC (Henan, China). They were incubated in RPMI 1640 medium (Gibco, Shanghai, China) supplemented with 10% FBS (BIOIND, Israel) and 1% penicillin‐streptomycin (PS) (100 U/mL penicillin and 100 μg/mL streptomycin) in a humidified incubator at 37°C with 5% CO_2_.

### 2.3 Cell Transfection

Cells were transfected with SORT1 small interfering RNA (siRNA) or its control (Ribo, Jiangsu, China), miR‐146a mimic or its control (GenePharma, Shanghai, China) using lipofectamine 3000 (Invitrogen by Thermo Fisher Scientific, Lithuania) following the manufacturer's recommendations. The sequence of SORT1 siRNA was 5′‐GCACAATCTTTACCTCAGA‐3′.

### 2.4 Reverse Transcription‐Quantitative PCR

Total RNA was extracted from the cells using the RNA simple Total RNA Kit (Tiangen, Beijing, China). The concentration and purity of the RNA were assessed using a microvolume spectrometer (Titertek Berthold, Germany). A total of 500 ng of the extracted RNA was subjected to reverse transcription to generate complementary DNA (cDNA) utilizing the PrimeScript RT Reagent Kit with gDNA Eraser (TaKaRa, Dalian, Liaoning, China). Following this, quantitative reverse transcription polymerase chain reaction (qRT‐PCR) analysis was conducted using the TB Green Premix Ex Taq II (TaKaRa, Dalian, Liaoning, China) within the QuantStudio 5 real‐time PCR system (Thermo Fisher Scientific).

MiRNAs were isolated utilizing the miRcute miRNA Isolation Kit (Tiangen, Beijing, China). cDNA for miRNAs was synthesized according to the manufacturer's protocol using the TransScript miRNA First‐Strand cDNA Synthesis SuperMix (TransGen Biotech, Beijing, China). The qRT‐PCR was performed employing the PerfectStart Green qPCR SuperMix (TransGen Biotech). The relative expression levels of mRNA and miRNA were assessed using the 2^−ΔΔCT^ method [20], with normalization to GAPDH for mRNA and U6 small nuclear RNA (snRNA) for miRNA.

The specific PCR primers utilized in this study were as follows: SORT1 forward (5′‐GAAGTCGTGGAGGAAGAATCTTT‐3′) and reverse (5′‐TGGTGTTGTCTGATCCCCATT‐3′); GAPDH forward (5′‐GTCATCCCTGAGCTGAACGG‐3′) and reverse (5′‐GGGTCTTACTCCTTGGAGGC‐3′); miR‐146a forward (5′‐TGAGAACTGAATTCCATGGGTT‐3′) and reverse (5′‐GATCGCCCTTCTACGTCGTAT‐3′); and U6 forward (5′‐CTCGCTTCGGCAGCACA‐3′) and reverse (5′‐AACGCTTCACGAATTTGCGT‐3′).

### 2.5 Western Blot

Cells were lysed utilizing a mixture of RIPA buffer (Solarbio, Beijing, China) and phenylmethylsulfonyl fluoride (PMSF) (Solarbio, Beijing, China) (100:1). The concentration of the extracted cellular proteins was quantified using a BCA protein assay kit (Sangon Biotech, Shanghai, China), followed by separation via acrylamide gel electrophoresis with a loading of 50 μg of protein per lane. Post‐electrophoresis, the proteins were transferred onto a PVDF membrane (Merck, Ireland), which was subsequently blocked with 5% non‐fat dry milk for 1.5 h. The membranes were then incubated overnight at 4°C with primary antibodies, followed by a one‐hour incubation at room temperature with horseradish peroxidase‐conjugated secondary antibodies. The antibodies utilized included rabbit anti‐SORT1 (1: 1000, Proteintech, Wuhan, China), GAPDH (1: 5000, Sangon Biotech, Shanghai, China), and HRP‐conjugated goat anti‐rabbit IgG (1: 5000, Sangon Biotech, Shanghai, China). The protein bands were subsequently visualized utilizing an enhanced chemiluminescence reagent (Thermo Fisher Scientific), and the relative expression levels of the target proteins were normalized to the intensity of the GAPDH bands, providing a reliable assessment of protein expression within the samples.

### 2.6 Cell Counting Kit‐8 (CCK‐8) Assay

NSCLC cells were seeded at a density of 3 × 10^3^ cells per well into 96‐well plates and incubated for 4 h. Following this initial incubation period, the cells were treated with SORT1 siRNA, miR‐146a mimic, or their controls. These treatments were conducted over 24, 48, 72, or 96 h at 37°C. Subsequently, 10 μL of CCK‐8 reagent (Biosharp, Beijing, China) was added to each well, and the plates were incubated for an additional hour. The optical density was assessed at a wavelength of 450 nm utilizing a multifunctional enzyme‐labeled instrument (PerkinElmer, Shanghai, China).

### 2.7 Cell Apoptosis Assay

Cell cultures were subjected to two washes with cold PBS buffer and resuspended in 1× binding buffer at a density of 10^5^ cells per 100 μL. Following this, 5 μL of FITC Annexin V (BD Biosciences, San Jose, CA, USA) and 5 μL of Propidium Iodide Staining Solution were added to the cell suspension. The cells were then incubated in the dark at room temperature for 15 min. Data from the assay were analyzed using flow cytometry (BD Biosciences, San Jose, CA, USA), allowing for the assessment of apoptosis.

### 2.8 Scratch Wound Assay

For the assessment of cellular migration, NSCLC cells were seeded at a density of 10^6^ cells per well in six‐well plates and allowed to adhere for 4 h. Following this incubation period, a standardized scratch was made across the cell monolayer using a sterile 200 μL pipette tip, and any detached cells were subsequently removed by washing with a serum‐free medium to ensure a clear wound margin. The NSCLC cells were then maintained in serum‐free RPMI‐1640 medium for 24 h. Imaging of the wounded areas was conducted at both time points: 0 h (immediately after scratching) and 24 h post‐scratch. The degree of migration was quantitatively determined by calculating the migration area using the formula migration area (%) = [(A1−A2)/A1] × 100%, where A1 represents the initial wound area at 0 h and A2 denotes the wound area at 24 h.

### 2.9 Transwell Assay

NSCLC cells, following transfection with SORT1 siRNA or its control, miR‐146a mimic or its control, were cultured in the upper chamber of a transwell insert, which was pre‐treated with matrigel (ABW, Shanghai, China; note that the migration assay was performed without the application of matrigel). Concurrently, 700 μL of RPMI‐1640 medium supplemented with 10% FBS was added to the lower chamber. After a 24 h incubation at 37°C, non‐migratory cells in the upper chamber were removed using a moistened cotton swab. Subsequently, the migratory cells that had traversed to the lower chamber were stained with 0.1% crystal violet and visualized using an inverted fluorescent microscope (Olympus, Tokyo, Japan).

### 2.10 Dual‐Luciferase Reporter Gene Assay

We used the TargetScan database (https://www.targetscan.org/vert_80/) to predict the relationship between SORT1 and miR‐146. A dual luciferase reporter vector was constructed by inserting SORT1, both wild‐type (Wt) and mutant (Mut) forms, encompassing a putative binding site for miR‐146a, into the pmiRGLO vector. The constructs pmiRGLO‐SORT1‐Wt or pmiRGLO‐SORT1‐Mut, miR‐146a mimics or its control were subsequently transfected into H‐1299 lung cancer cells using lipofectamine 3000. Twenty‐four hours later, luciferase activity was quantified using the Dual‐Luciferase Reporter Assay System (Promega, Madison, USA). The results were expressed as the ratio of firefly luciferase activity to renilla luciferase activity.

### 2.11 Immunofluorescence

The H1299 cells were immobilized in 4% paraformaldehyde at room temperature for 15 min to fix cellular morphology. H1299 cells were then permeabilized using 0.2% Triton X‐100 for 10 min to enhance antibody accessibility. After a blocking step to minimize non‐specific binding, which lasted 1 h, the cells were incubated overnight at 4°C with an anti‐Ki67 antibody (KeyGen, Jiangsu, China). Subsequently, the cells were treated with FITC‐conjugated goat anti‐rabbit IgG (KeyGen, Jiangsu, China) to facilitate detection. After the appropriate incubation period, the specimens were visualized using an inverted fluorescent microscope (Olympus, Japan) to capture images for further analysis.

### 2.12 Statistical Analysis

Data were processed using SPSS version 26.0 statistical software (IBM, Armonk, NY, USA). The results of the data measurements are expressed as mean and standard deviation. The independent samples *t*‐test was employed to compare the means between two independent groups. For the assessment of means across multiple groups, a one‐way analysis of variance (ANOVA) was applied. A *p*‐value of less than 0.05 was deemed statistically significant.

## 3 Results

### 3.1 SORT1 Protein was Highly Expressed in NSCLC

**FIGURE 1:**
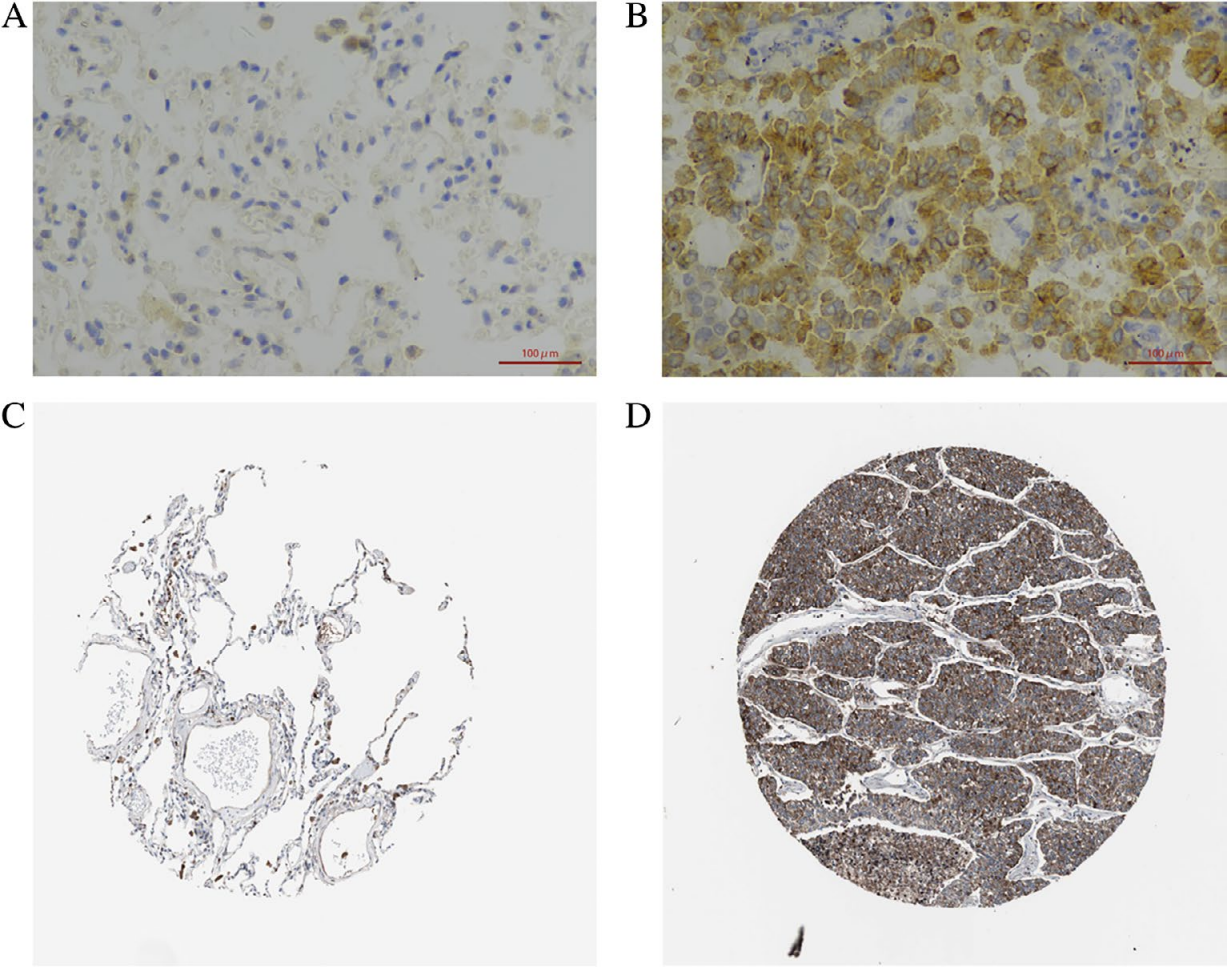
SORT1 was upregulated in the tumor tissue of NSCLC. (×400). (A) was adjacent non‐cancerous tissue, and (B) was tumor tissue from our clinical specimen; (C) was lung tissue, and (D) was tumor tissue from healthy individuals. Both (C, D) were from the human protein atlas database.)

We utilized immunohistochemistry to evaluate the expression levels of SORT1 in both cancerous tissues and adjacent non‐cancerous tissues obtained from patients diagnosed with lung adenocarcinoma. Our results demonstrated a significant upregulation of SORT1 expression within the lung adenocarcinoma specimens, predominantly localized to the cancer cells, as illustrated in Figures 1A,B. In addition, we also searched the Human Protein Atlas database and found that SORT1 was increased in lung adenocarcinoma (Figure 1C,D).

### 3.2 Inhibition of SORT1 Reduced Proliferation and Promoted Apoptosis in NSCLC Cells

**FIGURE 2:**
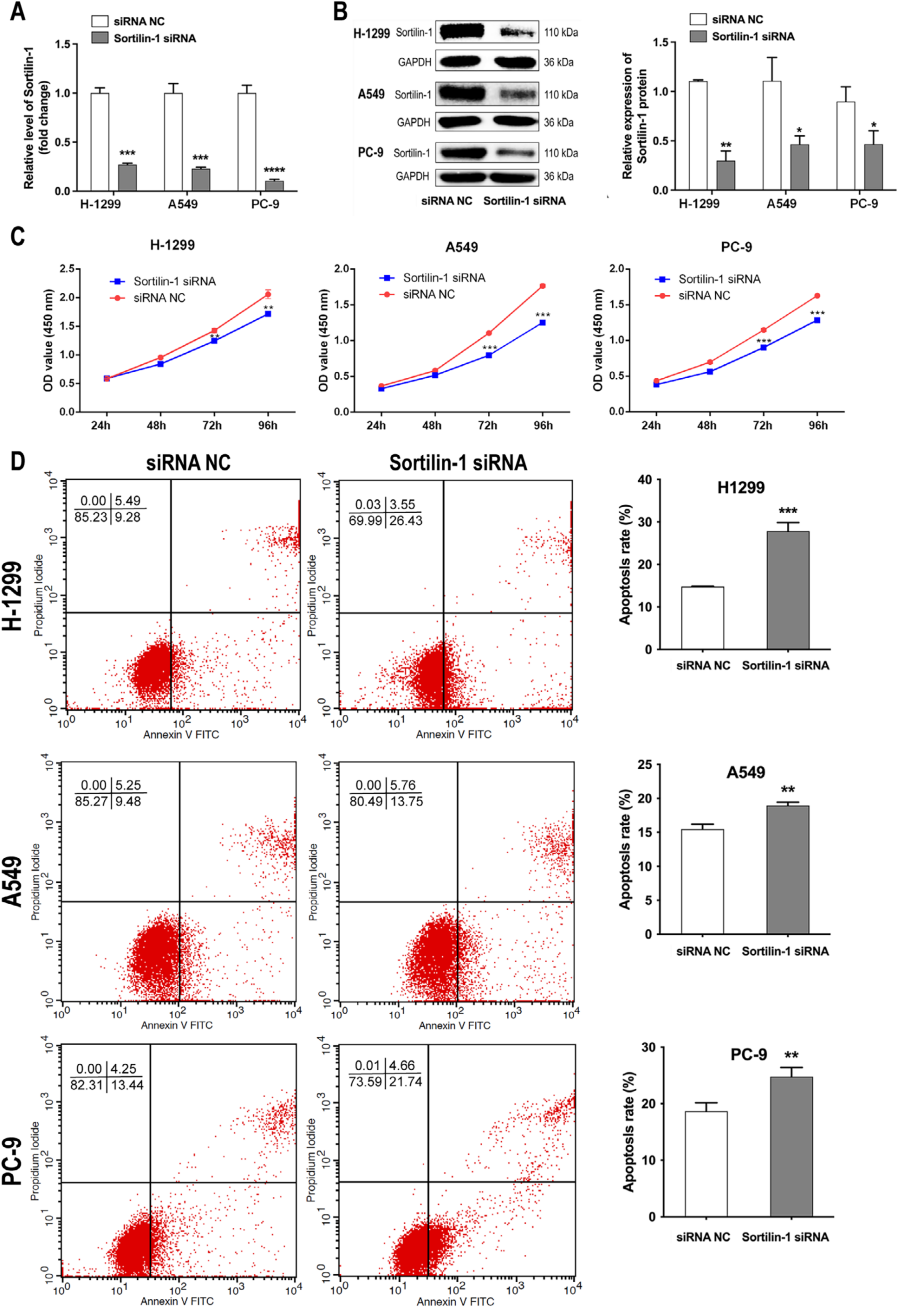
Inhibition of SORT1 significantly reduced the proliferation of NSCLC cells and enhanced apoptosis. (A) The expression levels of SORT1 in NSCLC cells subjected to transfection with SORT1 siRNA or control were quantified using RT‐qPCR. (B) The SORT1 protein in NSCLC cells treated with SORT1 siRNA or control was assessed through western blot analysis. (C) Cell viability and (D) apoptotic rates in NSCLC cells transfected with SORT1 siRNA or control were evaluated using the CCK‐8 assay and flow cytometry. Data in A, B, C and E indicate mean and SD from three separate experiments. Statistical significance was established at *p < 0.05, **p < 0.01, ***p < 0.001, ****p < 0.0001 when compared to the control groups.

Next, we investigated the functional impact of SORT1 in NSCLC cells using siRNA. As shown in Figure 2A,B, the mRNA and protein levels of SORT1 were significantly down‐regulated in H‐1299, A549, and PC‐9 cell lines by SORT1 siRNA. Furthermore, SORT1 siRNA inhibited the proliferation of NSCLC cells (Figure 2C). In addition, SORT1 siRNA exhibited apoptosis‐inducing effects in NSCLC cells (Figure 2D). Overall, these data indicate that SORT1 siRNA significantly promote apoptosis and inhibit cell survival in NSCLC cells.

### 3.3 Inhibition of SORT1 Significantly Impaired the Migration and Invasion of NSCLC Cells

**FIGURE 3:**
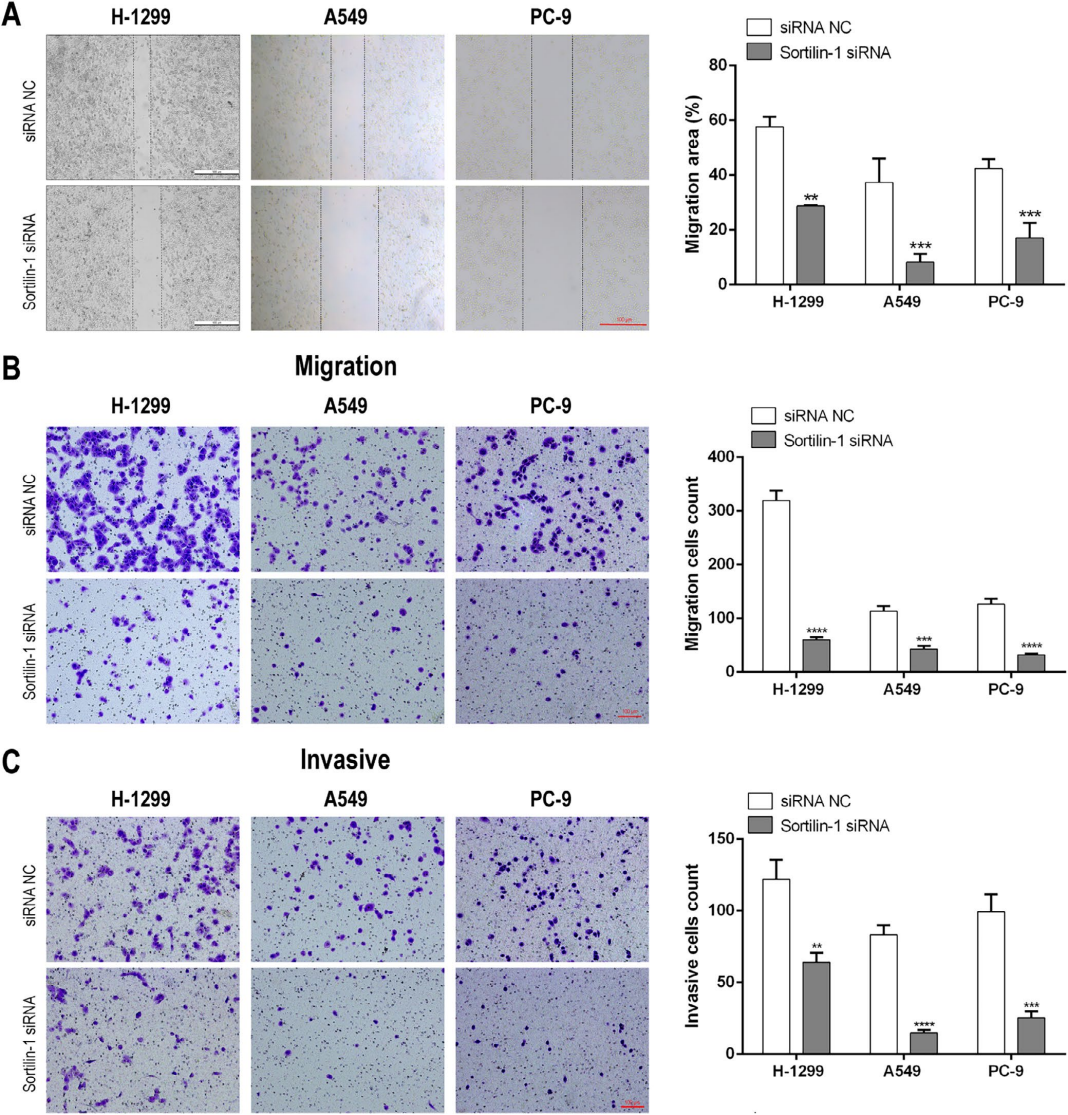
Inhibition of SORT1 significantly decreased migration and invasiveness of NSCLC cells. NSCLC cells were transfected with SORT1 siRNA or its control, and the migratory and invasive potentials were determined by (A) wound assay (scale bar = 100 μm), (B) transwell assay with (B) or without matrigel (C) (scale bar = 100 μm). Data in A, B, and C indicate mean and SD from three separate experiments. Statistical significance was determined with **p < 0.01, ***p < 0.001, and ****p < 0.0001 when compared to the siRNA NC group.

To evaluate the impact of SORT1 on the migratory and invasive capabilities of NSCLC cells, a scratch wound assay and a transwell assay were performed. We found a marked reduction in the migration of NSCLC cells following SORT1 siRNA treatment (Figure 3A). Additionally, the transwell assay revealed a substantial decrease in migrating and invasive NSCLC cells after 24 h of treatment with SORT1 siRNA compared to the control group (Figure 3B,C). Collectively, these findings provide evidence that SORT1 regulates the migration and invasiveness of NSCLC cells.

### 3.4 SORT1 Was a Direct Target of miR‐146a

**FIGURE 4:**
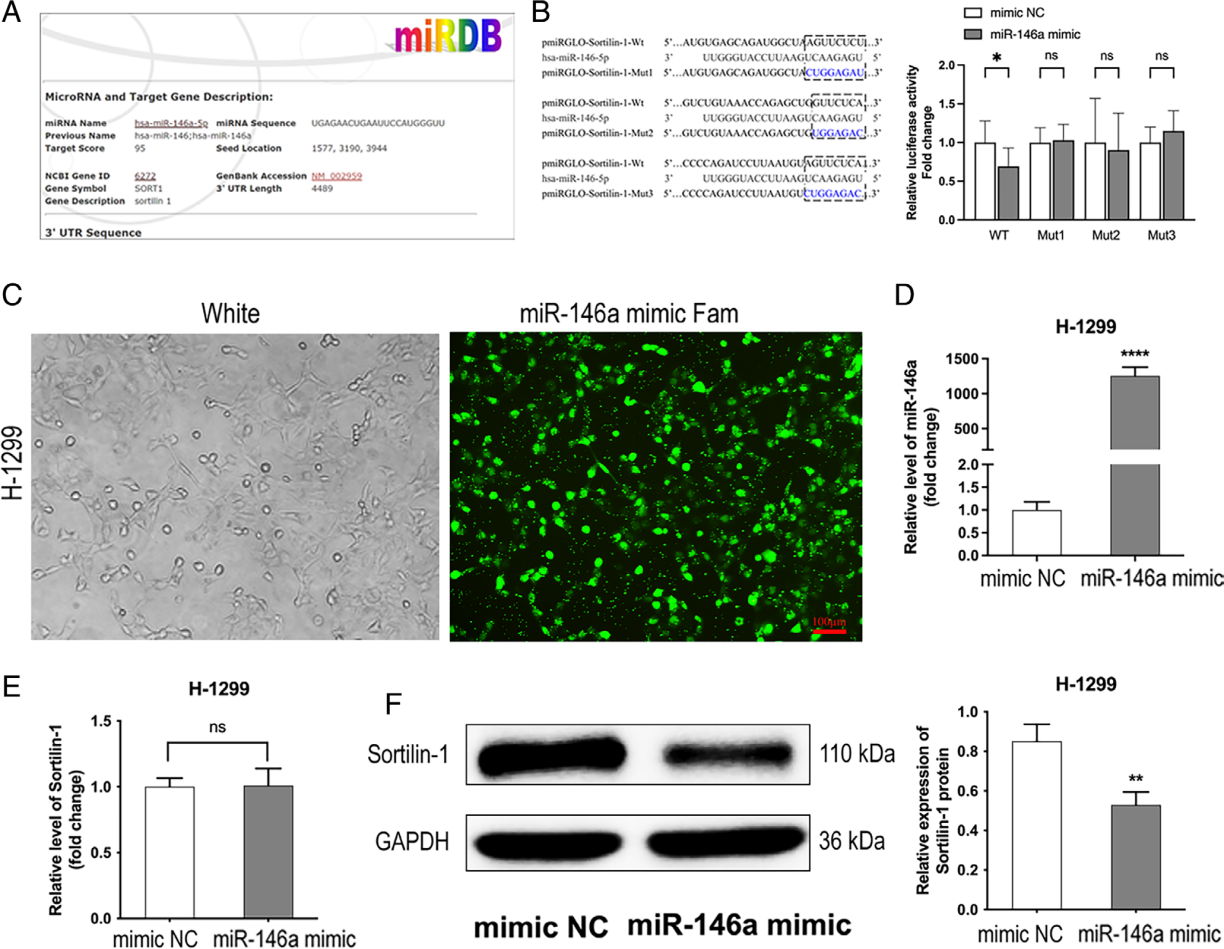
SORT1 was a direct target of miR‐146a. (A) SORT1 was a direct target of miR‐146a, predicted by targetscan. (B) H1299 cells were cotransfected with miR‐146a mimic or its control and pmiRGLO‐SORT1‐Wt or pmiRGLO‐SORT1‐Mut; the relative luciferase activity was detected by dual luciferase reporter assay. (C) Fluorescence staining analysis of Fam labeled miR‐146a mimic in H‐1299 cells (Scale bar = 100 μm). (D) The expression of miR‐146a in H‐1299 cells transfected with miR‐146a mimic or control was tested by RT‐qPCR. (E) The expression of SORT1 in H‐1299 cells transfected with miR‐146a mimic or control was tested by RT‐qPCR. (F) The protein levels of SORT1 in H‐1299 cells transfected with miR‐146a mimic or control were tested by Western blot. Data in B, D, E and F indicate mean and SD from three separate experiments. **p < 0.01, ***p < 0.001 compared to mimic NC. Ns, not significant.

We utilized TargetScan to ascertain miRNAs capable of regulating SORT1, leading us to hypothesize that SORT1 may serve as a potential target for miR‐146a (Figure 4A). Introducing a miR‐146a mimic resulted in a statistically significant decrease in relative luciferase activity in cells transfected with reporter vectors containing pmiRGLO‐SORT1‐Wt. In contrast, no significant effects were observed in cells harboring the mutant form, pmiRGLO‐SORT1‐Mut (Figure 4B). Furthermore, we introduced miR‐146a mimic‐Fam into H‐1299 cells (Figure 4C). RT‐qPCR analysis demonstrated a pronounced increase in miR‐146a expression levels in H‐1299 cells transfected with the miR‐146a mimic (Figure 4D). The levels of SORT1 mRNA (Figure 4E) remained unchanged in response to the miR‐146a mimic; however, the expression of SORT1 protein (Figure 4F) in H‐1299 cells decreased following transfection with the miR‐146a mimic. These findings present evidence that miR‐146a exerts its regulatory effects on SORT1 by downregulating its protein expression by inhibiting mRNA translation rather than through mRNA degradation.

### 3.5 MiR‐146a Promoted Apoptosis and Inhibited the Proliferation of H‐1299 Cells

**FIGURE 5:**
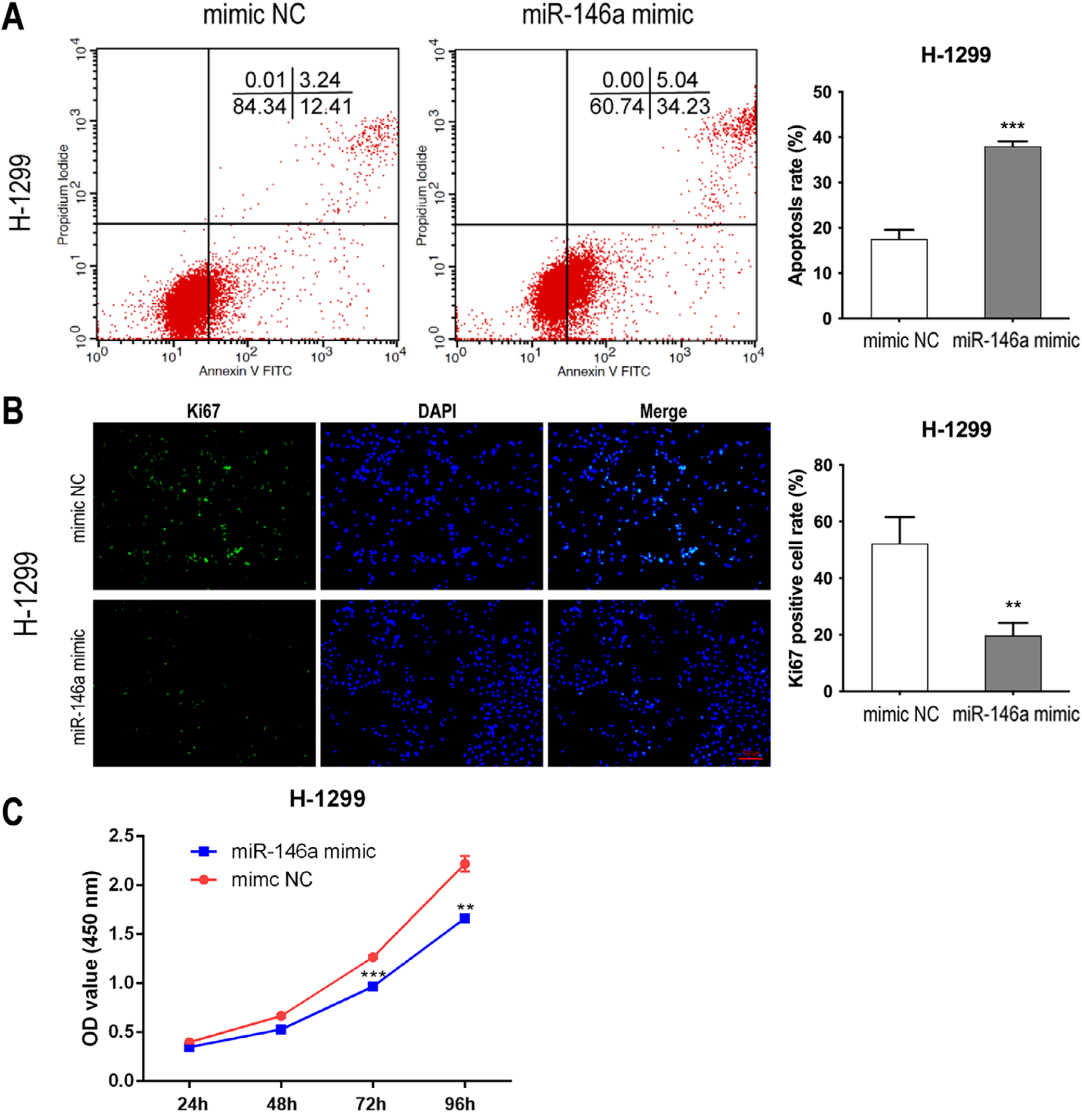
MiR‐146a promoted apoptosis and inhibited the proliferation of H‐1299 cells. (A) The apoptosis of H‐1299 cells transfected with miR‐146a mimic or control was tested by Annexin V/PI assay. The proliferation of H‐1299 cells transfected with miR‐146a mimic or control was tested by Ki67 staining (scale bar = 100 μm) (B) or CCK‐8 assay (C). Data in A, B and C indicate mean and SD from three separate experiments. **p < 0.01, ***p < 0.001 compared to mimic NC.

The Annexin V/PI assay revealed that miR‐146a mimic significantly increased the apoptosis of H‐1299 cells (Figure 5A). In addition, miR‐146a mimic suppressed the proliferation of H‐1299 cells, as indicated by Ki67 (Figure 5B) and CCK‐8 assays (Figure 5C).

### 3.6 MiR‐146a Significantly Inhibited the Migration and Invasion of H‐1299 Cells

**FIGURE 6:**
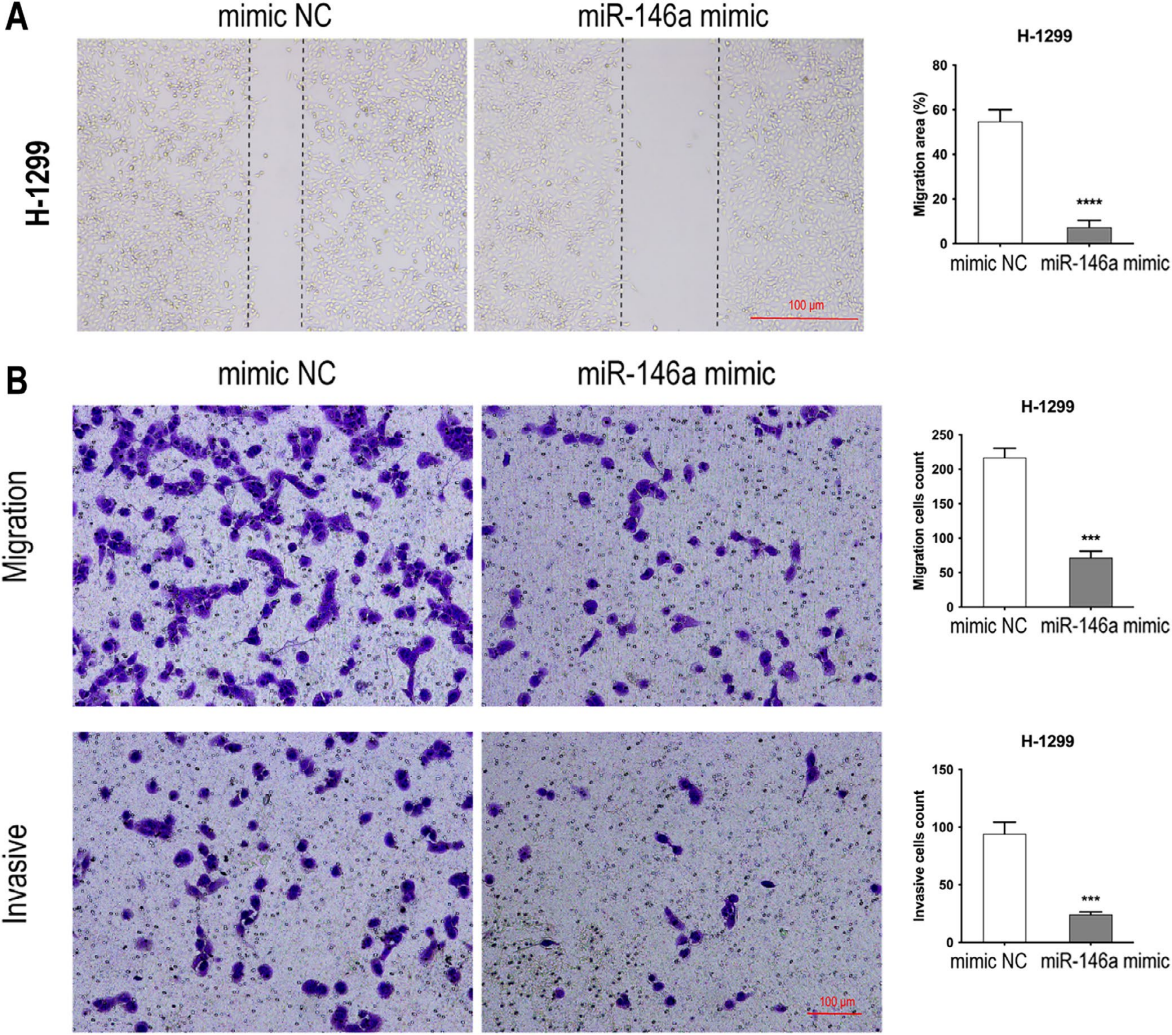
MiR‐146a significantly inhibited the migration and invasion of H‐1299 cells. H1299 cells were transfected with miR‐46a mimics or controls; wound assay (A) and transwell were used to test the migration and invasion (B) of H1299 cells (scale bar = 100 μm). ***p < 0.001, ****p < 0.0001 compared to the control.

The transfection of H‐1299 cells with a miR‐146a mimic led to a significant reduction in cellular migration (Figure 6A). Furthermore, the transwell assay corroborated these findings, demonstrating that the miR‐146a mimic effectively inhibited not only the migration but also the invasive capacity of H‐1299 cells (Figure 6B). Collectively, these results suggest that miR‐146a plays a critical role in impeding the migratory and invasive properties of H‐1299 cells.

## 4 Discussion

The key findings of this investigation are as follows: Firstly, SORT1 exhibited significant upregulation in tumor tissue derived from NSCLC. Secondly, inhibiting SORT1 resulted in a reduction in the proliferation, migration, and invasiveness of NSCLC cells in vitro, while also promoting apoptotic processes. Thirdly, SORT1 was a target of miR‐146a. Fourthly, miR‐146a demonstrated effects that enhanced apoptosis and impeded the proliferation, migration, and invasion of NSCLC cells in vitro. Collectively, these findings suggest that miR‐146a may diminish the oncogenic potential of NSCLC through the targeted modulation of SORT1.

The pathogenetic role of SORT1 in various cancers, including breast [8, 21], colorectal [22], and gastric [23] malignancies, has been investigated; however, its role in NSCLC remains largely unexplored. Recent research has demonstrated that SORT1, in conjunction with TrkB and EGFR, facilitates the release and transfer of extracellular vesicles from NSCLC cell lines via endocytosis [24]. These extracellular vesicles, characterized by the presence of the SORT1‐EGFR‐TrkB complex (hereafter referred to as the TES complex), can be internalized by recipient cells, promoting angiogenesis and enhancing cellular proliferation, survival, adhesion, and migration of NSCLC cells [24]. Additionally, SORT1 regulates EGFR internalization from the plasma membrane, thereby constraining proliferative signaling that drives tumor aggressiveness [11]. A previous study utilizing RT‐PCR indicated that SORT1 mRNA levels were decreased in lung cancer tissue [25]. However, this study did not employ western blotting or immunohistochemistry to detect SORT1 protein levels. In contrast, the current study used immunohistochemistry and found that SORT1 was increased in lung cancer tissue. This discrepancy has some possible explanations. First, SORT1 may undergo post‐transcriptional modifications, such as those involving miRNAs. Second, the composition of cancer tissue is complex and includes various cell types, such as tumor cells, immune cells, tumor‐associated fibroblasts, and endothelial cells. Therefore, while SORT1 mRNA may be elevated in cancer cells, the overall expression level in the cancer tissue could be reduced due to its lower expression in non‐cancerous cells within the tumor microenvironment. Furthermore, the influence of SORT1 on apoptosis, migration, and invasiveness in lung cancer, as well as the miRNAs that regulate SORT1, has yet to be comprehensively examined. In our study, we observed that SORT1 expression was significantly upregulated in lung cancer tumor tissues, a finding that aligns with earlier studies identifying increased SORT1 levels in other cancer types, including breast [8], ovarian [9], and liver [26] cancers. Our experimental results indicate that the knockdown of SORT1 reduces the proliferation, migration, and invasiveness of lung cancer cells in vitro, while simultaneously promotes apoptosis. These results suggest that SORT1 may function as a tumor promotor in this context. This assertion is further substantiated by previous investigations that correlate elevated SORT1 levels with poor prognostic outcomes in liver [26] cancer and glioblastoma [27]. Collectively, these findings indicate SORT1 as a promising therapeutic target for NSCLC.

A notable strength of our study lies in the identification of miR‐146a as a regulatory factor for SORT1 and its subsequent influence on apoptosis, invasiveness, and proliferation in lung cancer through the modulation of SORT1 expression. Previous investigations have documented that lung cancer cell lines, such as those referenced in A549, H1299, and H1975, exhibit decreased levels of miR‐146a in comparison to benign cell lines, specifically Beas2B [28, 29]. Furthermore, analyses of lung cancer tissue have revealed decreased levels of miR‐146a relative to normal lung tissue [29, 30, 31], with a progressive decline in miR‐146a expression corresponding to advancing stages of lung cancer [31]. Importantly, increased levels of miR‐146a have been correlated with improved prognostic outcomes in lung cancer patients [31, 32], reinforcing the notion that miR‐146a functions as a tumor suppressor. In vitro studies have substantiated the role of miR‐146a in inhibiting cellular proliferation [33], enhancing sensitivity to chemotherapeutic agents [33], and reducing migration and invasiveness via the targeting of various oncogenic pathways, including CCNJ [34], Merlin [35], EGFR [36], CCND1 [29], CCND2 [29], and TCSF [37]. Our findings elucidate a novel pathway elucidating the pathogenetic contributions of miR‐146a in NSCLC, specifically through its targeting of SORT1. The regulatory influence of miR‐146a on SORT1 has previously been implicated in other pathological conditions, including atherosclerosis [38] and cerebral ischemia/reperfusion injury [39].

While this study represents the inaugural investigation into the miR‐146a/SORT1 axis and its impact on the oncologic behavior of NSCLC, several limitations must be acknowledged. Firstly, the findings have not been corroborated by in vivo studies, which restrict the generalizability of the results. Additionally, we discovered that miR‐146a can regulate the expression of SORT1 by binding to specific regions of its mRNA. Both miR‐146a and SORT1 play a role in influencing the phenotype of cancer cells. However, the molecular mechanisms through which SORT1 impacts the phenotypic characteristics of NSCLC cancer cells are not yet fully understood. Further studies are necessary to explore this matter.

In summary, our study demonstrates that SORT1, under the regulatory influence of miR‐146a, plays a crucial role in enhancing the oncogenic behaviors of NSCLC. Consequently, both miR‐146a and SORT1 emerge as promising novel therapeutic targets for NSCLC.

## Author Contributions

Xi Lin and Zhi Yan conducted most of the experiments, except for immunohistochemistry, and were responsible for drafting the initial manuscript. Ling Hai executed the immunohistochemistry procedures. Yan Niu, Jian‐Xun Wen, Hong‐Zhe Zhu, Cheng Yan, and Su‐Na Cha contributed valuable technical assistance throughout the study. Li Yan and Wen‐Qi Zheng engaged in revising the manuscript. Li Yan secured the funding necessary for the project. Man Zhang and Zhi‐De Hu were instrumental in designing and supervising the study and reviewing and editing the manuscript. All authors have consented to the submission of this work.

## Ethics Statement

This study was approved by the ethics committees of the Affiliated Hospital of Inner Mongolia Medical University (No: 2025004).

## Consent

Informed consent was waived because we retrospectively used residual materials for research.

## Conflicts of Interest

The authors declare no conflicts of interest.

## Data Availability

The data are not publicly available due to ethical restrictions.
